# One new species and three newly recorded species of *Neopallodes* Reitter from China (Coleoptera, Nitidulidae, Nitidulinae)

**DOI:** 10.3897/zookeys.880.38033

**Published:** 2019-10-14

**Authors:** XiaoXiao Chen, Min Huang

**Affiliations:** 1 Key Laboratory of Plant Protection Resources and Pest Management of the Ministry of Education, Entomological Museum, Northwest A&F University, Yangling, Shaanxi 712100, China Northwest A&F University Yangling China

**Keywords:** Chinese fauna, Cucujoidea, *Cyllodes*-complex, Cyllodini, distribution

## Abstract

One new species of the sap beetle genus *Neopallodes*, *N. nigrescens***sp. nov.**, is described and illustrated. New information on the distribution and illustrations of three species, *N. dentatus* Kirejtshuk, 1994, *N. falsus* Grouvelle, 1913 and *N. vietnamicus* Kirejtshuk, 1987, which are newly recorded from China, are also supplemented. A key to species of the genus *Neopallodes* from China is provided.

## Introduction

The genus *Neopallodes* Reitter, 1884 was proposed for three species from Japan (Reitter 1884). Grouvelle (1892, 1902, 1903) later described another three species in this group although this taxon was treated as a synonym by Grouvelle (1913a). However, C.T. Parsons (1943) regarded *Neopallodes* as a valid genus. Five species from Japan and one species from Chejudo Island, Korea, were recorded by Y. Kurosawa et al. (1985) and M. Chûjô and C.E. Lee (Chûjô and Lee 1992), respectively. Kirejtshuk (1987, 1994, 2011) described 16 species and revised the Palaearctic and Indo-Malayan species. Later, Kirejtshuk (1992) distinguished *Neopallodes* from *Pallodes* Erichson, 1843, based on the exposed male anal sclerite from under the transverse pygidial apex, and proposed some additional diagnostic characters for these taxa. Twenty-one species previously placed in *Pallodes* Erichson, 1843 by Grouvelle (1896, 1906) and Reitter (1877) were also transferred to *Neopallodes* by Kirejtshuk (1994). Kirejtshuk (2008) gave a complete species checklist of *Neopallodes*, including 40 East Asian species (mostly in the Palaearchearctic or East-Chinese Province of the Palearctic Region, Indo-Malayan, and Malgassy regions.

Prior to our studies, only three species, *Neopallodes hilleri* Reitter, 1877, *N. inermis* Reitter, 1884, and *N. vicinus* Grouvelle, 1892, have been recorded from China (Kirejtshuk 1992, 1994). Here, we describe one new species and newly record three species from China. A key to the Chinese species of *Neopallodes* is presented.

## Materials and methods

All materials for this study are deposited in the Entomological Museum of Northwest A&F University (**NWAFU**), Yangling, China. Most samples were preserved in 99% ethanol, although some were preserved as dried specimens. All photographs were taken using a Leica M205A microscope with a Leica DFC camera, and image stacking was done using LAS (Leica Application Suite) V3.7. Images were retouched with Adobe Photoshop CS6. Illustrations were drawn using Adobe Illustrator CS4.

Morphological terminology follows Kirejtshuk (1994, 2011). Body length measures from the anterior edge of clypeus to the posterior apex of pygidium; body width refers to the maximum width of elytra.

## Taxonomy

### Neopallodes

Taxon classificationAnimaliaColeopteraNitidulidae

Reitter, 1884

C6AE4528-E69F-5E0A-BBCA-6DBB15B1873B

Neopallodes Reitter 1884: 269; Grouvelle 1892: 849, 1902: 17; Kirejtshuk 1987: 152; 1994: 225; 2008: 119; 2011: 287. Type species: Pallodes hilleri Reitter, 1877 (subsequent designation by Kirejtshuk 1994)

#### Diagnosis.

The genus *Neopallodes* can be distinguished from other genera of the *Cyllodes* complex by the following features: distance between metacoxae wider than that between procoxae and mesocoxae; tarsomeres simiple on all legs; male anal sclerite exposed from under truncate pygidial apex. This genus is similar to other cyllodin genera in East Asia, such as *Pallodes*, *Coxollodes*Kirejtshuk 1987, and *Cyllodes* Erichson 1843, but it can be easily distinguished from *Pallodes* and *Coxollodes* in having the exposed male anal sclerite from under the transverse pygidial apex, and from *Cyllodes* in having simple tarsomeres and axillary sclerites absent on the mesoventrite.

#### Remarks.

Congeners of *Neopallodes* are mycophagous, and their adults are associated with the sporocarps and thalli of Agaricaceae (Basidiomycetes). So far, larvae are known to be found on the mycelia of these fungi or in their fruiting bodies (Hayashi 1978; Kirejtshuk 1994; Leschen 1999; Yamashita and Hiji 2007). Yunnan Province accounts for 90% of the wild mushroom species in China (Liu 2014), and, the species in this genus so far collected in China are all distributed in Yunnan Province. This suggests that the abundance of fungi may potentially harbor undiscovered species of this genus.

### Key to the species of *Neopallodes* from China^1^

**Table:** 

1	Dorsal surface without color spots (Figs 1, 3, 18)	**2**
–	Dorsal surface with dark spots (Figs 19, 20)	**4**
2	Outer apical angle of protibia without distinctly raised tooth (Fig. 9); scutellum subtriangular with round apex (Figs 1, 3); prosternum slightly carinate with moderately subflattened process slightly widened at subtruncate apex and not bend to mesoventrite between mesocoxa; abdominal ventrite 1 much longer than hypopygidium (Figs 2, 4)	**3**
–	Outer apical angle of protibia with distinctly raised tooth; scutellum trapezoidal (Fig. 18); prosternum strongly carinate with process extremely widened at round apex and bend to mesoventrite between mesocoxa; abdominal ventrite 1 scarcely longer than hypopygidium	***N. dentatus* Grouvelle, 1892**
3	Base of pronotum nearly three times as wide as long; antennal club subequal with or wider than prosternal process, antennal club subequal in length with antennomeres 2–8 together, antennomere 11 about as long as wide, antennomere 9 subequal in length with antennomere11; metaventrite without punctures in the middle and with fine and sparse punctures laterally	***N. inermis* (Reitter, 1885)**
–	Base of pronotum nearly 2.4 times as wide as long (Figs 1, 3); antennal club narrower than prosternal process; antennal club distinctly shorter than antennomeres 2–8 together, antennomere 11 wider than long, antennomere 9 shorter than antennomere11 (Fig. 8); metaventrite with fine punctures in the middle and large, sparse punctures laterally (Figs 2, 4)	***N. nigrescens* sp. nov.**
4	Outer apical angle of protibia without distinctly raised tooth; antennal club distinctly shorter than antennomeres 2–8 together; dorsal surface usually without color spots	***N. vicinus* Grouvelle, 1892**
–	Outer apical angle of protibia with distinctly raised tooth; antennal club longer than or subequal with antennomeres 2–8 together; dorsal surface usually with distinct, large, blackish spots	**5**
5	Prosternal process flat apically; pronotum without dark spots at base; elytral surface with not quite regular longitudinal rows of punctures	***N. hilleri* (Reitter, 1877)**
–	Prosternal process carinate apically; pronotum with distinct, dark spots at base; elytral surface with distinctly regular longitudinal rows of punctures	**6**
6	Antennal club longer than antennomeres 2–8 together; elytra with longitudinal rows of punctures not quite regular at basal third; elytra with large, blackish spots at humeral angles; pronotum with two subcircular, blackish spots; protibia with strongly raised tooth. (Fig. 20)	***N. vietnamicus* (Kirejtshuk, 1987)**
–	Antennal club subequal in length with antennomeres 2–8 together; elytra with clearly longitudinal rows of punctures, including their basal third; elytra without spots at humeral angles; pronotum with two subtriangular, blackish spots; protibia with moderately raised tooth. (Fig. 19)	***N. falsus* (Grouvelle, 1913)**

### Neopallodes nigrescens

Taxon classificationAnimaliaColeopteraNitidulidae

Chen & Huang sp. nov.

E1CEBC95-A88E-504A-871F-24935C23A16A

http://zoobank.org/C50B513E-2F59-4C59-9199-EF629E6C36F3

Figs 1–17

#### Type material.

***Holotype***, ♂: China: Yunnan Province, Chuxiong City, Zixi Mountain, 2450 m a.s.l., 13-VII-2018, Xiaoxiao CHEN (NWAFU). ***Paratypes***, 5♂♂: Yunnan Province, Dali City, Cangshan Mountain, 2610 m a.s.l., 25°39'51"N, 100°07'10"W, 05-VI-2018, Xiaoxiao CHEN (NWAFU); 1♂: Yunnan Province, Qujing City, Junzi Mountain, 2310 m a.s.l., 30-VII-2018, Xiaoxiao CHEN (NWAFU); 2♂♂: Yunnan Province, Qujing City, Yehuagou, 1980 m a.s.l., 05-VII-2018, Xiaoxiao CHEN (NWAFU); 3♂♂, 8♀♀: Same as holotype; 2♂♂: Guizhou Province, Bijie City, Shangdi Mountain, 2240 m a.s.l., 26°52'13"N, 104°18'02"W, 06-VII-2018, Xiaoxiao CHEN (NWAFU).

#### Description.

Body size (♂): length 2.7–3.7, width 2.1–3.1 mm.

**Figures 1–17.**
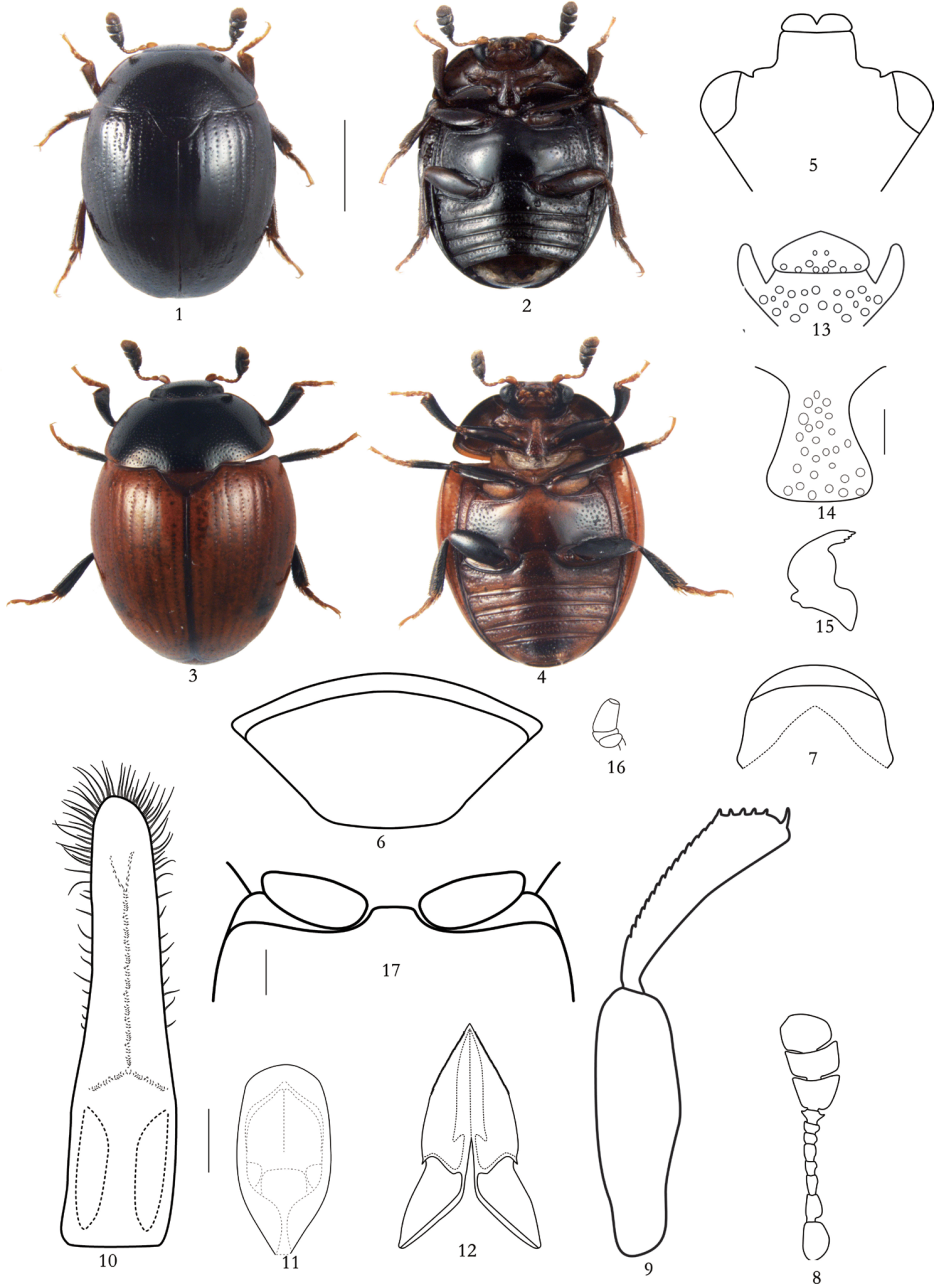
*Neopallodes nigrescens* sp. nov. (male) **1, 3** body, dorsal **2, 4** body, ventral **5** head, dorsal **6** pygidium **7** anal sclerite, ventral **8** left antenna, dorsal **9** left profemur and protibia, dorsal **10** tegmen, dorsal **11** penis trunk, dorsal **12** ovipositor, ventral **13** mentum and inner edges of antennal grooves, ventral **14** prosternal process, ventral **15** left mandible, dorsal **16** left maxillary palpus, ventral **17** anterior part of mesoventrite, ventral **1–2, 5–17** specimen from “Yunnan Province, Chuxiong City, Zixi Mountain” **3, 4** specimen from “Yunnan Province, Dali City, Cangshan Mountain” Scale bars: 1 cm (**1–4**); 0.2 cm (**5–12**); 0.2 cm (**13–16**); 0.2 cm (**17**).

***Body***: Body shiny, dorsum glabrous, abdomen with sparse and inconspicuous hairs, moderately convex dorsally and ventrally. Dorsal and ventral surface blackish with antennae and tarsi lighter, or dorsal surface blackish with elytra brightly brownish orange, ventral surface yellowish brown with metaventrite darker (Figs 1–4).

***Dorsal habitus***: Head somewhat depressed with medium-sized eyes, punctures larger than eye-facets. Lobes of labrum clearly exposed with short excision (Fig. 5). Mandible exposed from under lobes of labrum, with four small teeth on apical edge (Fig. 15). Length of antenna subequal with head width, scape subcylindrical, slightly curved and about 1.5 times as long as wide, pedicel approximately subcylindrical and nearly 1.5 times as long as wide, antennomere 3 narrowed basally, antennomeres 3 and 4 longer than wide, antennomere 4 longer than antennomere 5, each of antennomeres 5–8 wider than long, antennal club compact and asymmetrical, with length nearly 0.6 times of total antennal length, antennomere 11 shorter than antennomeres 9–10 together (Fig. 8). Pronotum widest at base and arcuately narrowed to apex; anterior edge deeply emarginate; posterior edge vaulted with clear projection covering base of scutellum, anterior and posterior angles blunt; surface with punctures round, slightly smaller than eye-facets and separated by 2.7–5.7 puncture diameters; interspaces smooth to alutaceous. Scutellum subtriangular with round apex, with punctures scattered and separated by 0.7–1.9 puncture diameters. Elytra about 0.9 times as long as wide together, widest at basal 1/3; surface with regular longitudinal rows of large punctures separated by 0.8–1.3 diameters in rows; rows separated from each other by 5.3–6.9 puncture diameters; interspaces microreticular, between rows of large punctures with irregular row of very fine and sparse punctures. Pygidium markedly wider than long, subtruncate at apex and with dense punctures subequal to those on pronotum (Fig. 6). Anal sclerite slightly wider than long (Fig. 7).

***Ventral habitus***: Terminal maxillary palpomere elongate and subconical (Fig. 16). Terminal labial palpomere narrowing apically. Mentum pentagonal with sparse, large punctures along posterior edge (Fig. 13). Antennal grooves strongly convergent behind mentum. Prosternum convex medially, with moderately subflattened process, slightly widened at subtruncate apex, and about 1.7 times as wide as scape. Mesoventrite moderately carinate. Metaventrite with finer punctures in middle and coarser punctures laterally. Metepisternum somewhat narrower than antennal club and with distinct, large punctures. Epipleura almost 0.6 times as wide as antennal club long. Abdominal ventrite 1 longest, about 1.6 times as long as hypopygidium and 2.1 times longer than each of ventrites 2–4. Hypopygidium rounded at apex. Submesocoxal line almost rectilinearly deviating from posterior edge of mesocoxal cavity (Figs 2, 4). Distance between metacoxae more than three times as great as that between procoxae, and about twice as great as that between mesocoxae.

Protibia artcuately curved, about as wide as antennal club, with rounded outer apical angle and with apical angle round (Fig. 9); meso- and metatibiae almost subtriangular and slightly narrower than protibia, both with rows of dense setae along outer edge. Femora 1.8 times as wide as corresponding tibiae. Metatarsus shorter than corresponding tibia.

***Male genitalia***: Tegmen narrow, strongly sclerotized and rounded apically, about 4.3 times as long as wide, with short setae disposed along middle of tegmen and forming an X-like figure; also with long setae along sides and at apex (Fig. 10). Penis trunk about 2.1 times as long as wide and 0.4 times long as tegmen, with apex widely rounded and with two wide apical lobes narrowed apically (Fig. 11).

***Female***: Body size: length 2.9–3.5, width 2.1–2.8 mm. Ovipositor moderately sclerotized, gonocoxal apex acuminate (Fig. 14).

#### Variability.

Some variation is observable in coloration and punctures. The holotype and paratypes from Chuxiong City, Zixi Mountain, and Qujing City, Yehuagou (all Yunnan Province) are subunicolorous black, while the paratypes from Dali City, Cangshan Mountain, and Qujing City, Junzi Mountain (all Yunnan Province) and from Bijie City, Shangdi Mountain (Guizhou Province) are blackish with brightly brownish elytra.

#### Diagnosis.

*Neopallodes nigrescens* can be distinguished from other species of the genus *Neopallodes* by its unique body color (dorsal surface blackish or with elytra brightly brownish orange), elytra with regular longitudinal rows of large punctures and not quite regular longitudinal rows of small punctures arranged alternately and tegmen with short setae forming an X-like figure. This taxon is similar to *N. inermis*, but differs from it in: antennal club distinctly shorter than antennomeres 2–8 combined; metaventrite with fine punctures in the middle and with large, sparse punctures laterally; tegmen with short setae forming an X-like figure; penis trunk with round apex.

#### Name derivation.

The specific epithet is derived from the Latin word “*nigrescens*” (= blackening) referring to the color of dorsal surface of most specimens.

### Neopallodes dentatus

Taxon classificationAnimaliaColeopteraNitidulidae

Grouvelle, 1892

572AF350-AB51-5A7F-B086-4DAAF7048E6C

Figs 18 , 21 , 22

Neopallodes dentatus Grouvelle 1892: 849
Neopallodes dentatus Kirejtshuk 1994: 230

#### Material examined.

26♂♂, 22♀♀, China: Yunnan Province, Chuxiong City, Zixi Mountain, 2450 m a.s.l., 25°00'59"N, 101°24'10"W, 13-VIII-2018, Xiaoxiao CHEN (NWAFU); 2♂♂: Shaanxi Province, Ziyang County, Fenghuang Mountain Bell and Drum Tower Scenic Area, 27-VI-2018, Yuru YANG (NWAFU).

#### Distribution.

China (Yunnan, Shaanxi), Myanmar

### Neopallodes falsus

Taxon classificationAnimaliaColeopteraNitidulidae

Grouvelle, 1913

41CF9D18-1845-5905-86EA-4357915A698E

Figs 19 , 23 , 24

Pallodes harmandi Grouvelle 1903:117, non Grouvelle 1902: 17; Grouvelle 1908: 392; Grouvelle 1913:169
Pallodes falsus Grouvelle, 1913b: 398
Neopallodes lindskogi Kirejtshuk 1987: 158
Neopallodes falsus Kirejtshuk 1994: 237

#### Material examined.

3♂♂, China: Yunnan Province, Yuxi City, Ailao Mountain, 2200 m a.s.l., 20-VII-2017, Xiaoxiao CHEN (NWAFU); 7♂♂, 6♀♀, China: Yunnan Province, Qujing City, Junzi Mountain, 2150 m a.s.l., 14-VII-2017, 30-VII-2018, Xiaoxiao CHEN (NWAFU).

#### Distribution.

China (Yunnan), India, Japan, Myanmar, Nepal

### Neopallodes vietnamicus

Taxon classificationAnimaliaColeopteraNitidulidae

Kirejtshuk, 1987

3B34A990-294A-5229-9034-B7A42248C3AB

Figs 20 , 25 , 26

Neopallodes vietnamicus Kirejtshuk 1987: 152.

#### Material examined.

1♂, China, Yunnan Province, Yuxi City, Mopanshan Forest Park, 2300 m a.s.l., 22-VI-2016, Meike LIU (NWAFU); 2♂♂, China, Yunnan Province, Yuxi City, Ailao Mountain, 2220 m a.s.l., 20-VII-2017, Xiaoxiao CHEN (NWAFU); 1♂, China: Yunnan, Qujing City, Junzi Mountain, 2300 m a.s.l., 14-VII-2017, Xiaoxiao CHEN (NWAFU).

#### Distribution.

China (Yunnan), India, Myanmar, Vietnam

**Figures 18–26.**
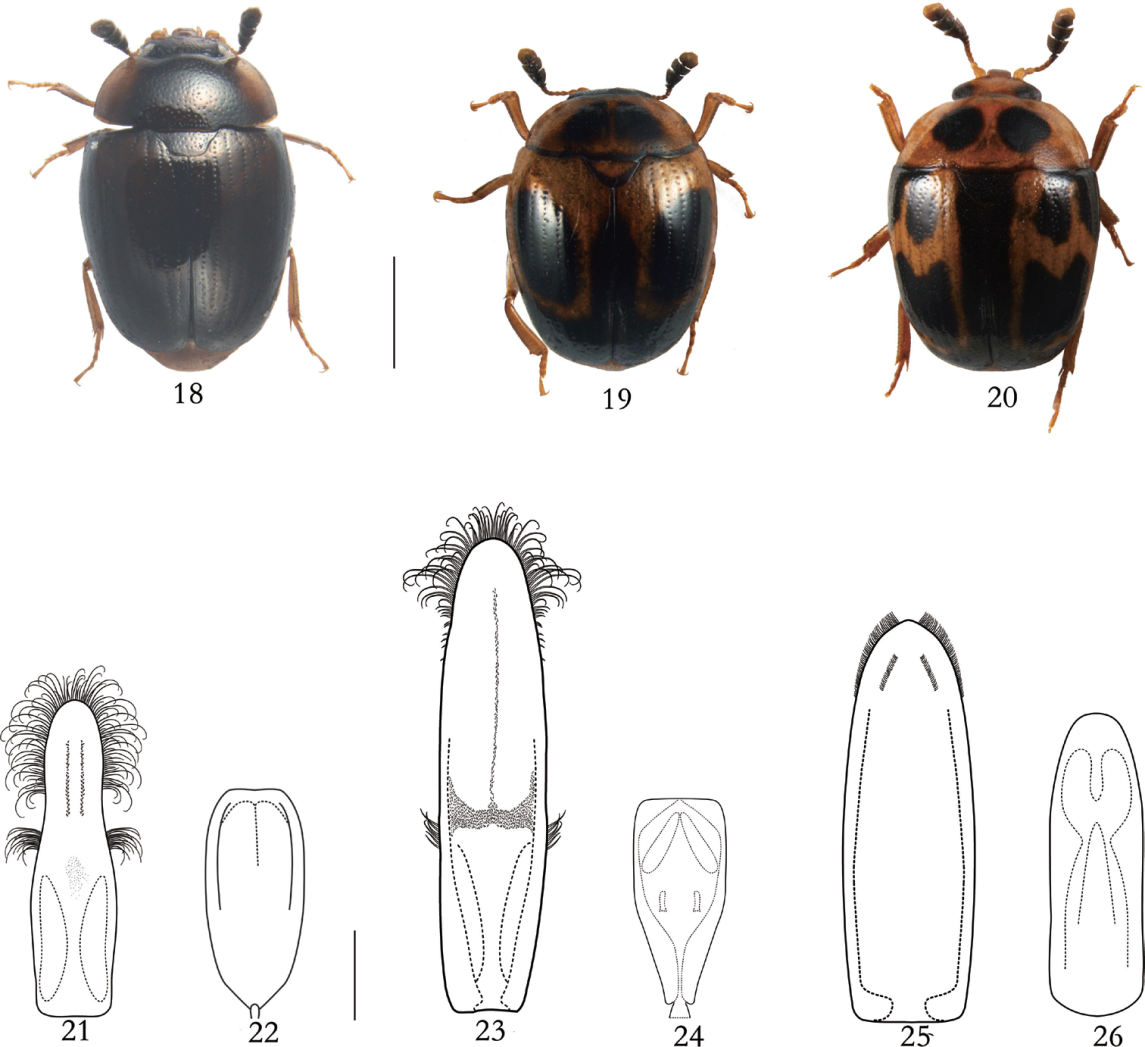
*Neopallodes dentatus*, *N. falsus*, and *N. vietnamicus***18, 21, 22***N. dentatus* (male): **18** dorsal view **21** tegmen, dorsal **22** penis trunk, dorsal. **19, 23, 24***N. falsus* (male): **19** dorsal view **23** tegmen, dorsal **24** penis trunk, dorsal. **20, 25–26***N. vietnamicus* (male): **20** dorsal view **25** tegmen, dorsal **26** penis trunk, dorsal. Scale bars: 1 cm (**18–20**); 0.2 cm (**21–26**).

## Supplementary Material

XML Treatment for Neopallodes

XML Treatment for Neopallodes nigrescens

XML Treatment for Neopallodes dentatus

XML Treatment for Neopallodes falsus

XML Treatment for Neopallodes vietnamicus

## Footnotes

after Kirejtshuk 1994.
